# Supplementary material for: Content-rich biological network constructed by mining PubMed abstracts
Source: BMC Bioinformatics. 2004 Oct 8;5:147. doi: 10.1186/1471-2105-5-147 (PMC528731; doi:10.1186/1471-2105-5-147)
Supplement: Additional File 5 — The original Chilibot query results of the term "long-term potentiation (LTP)" and 22 other terms, limiting the latest references analyzed to the years 1990, 1995, 2000, and 2004. [file 1471-2105-5-147-S5.bz2 › chilibotAdditionalFile5/ltp2000/html/SYNAPSIN I_CAMKIV.html]

 


 **SYNAPSIN I** and **CAMKIV** 
  
Found 1 abstracts in PubMed,  **1 abstracts were retrieved and analyzed**.  


---

 Search Google  |
 PDF files only 
|  EDU domain only 

---

- J Biol Chem, 1998   **Definition of optimal substrate recognition motifs of Ca2ion calmodulin dependent protein kinases IV and II reveals shared and distinctive features.** .
  The substrate recognition determinants of Ca2ion calmodulin dependent protein kinase CaMK IV and CaMKIIalpha were investigated using peptide substrates modeled on the amino acid sequence encompassing Ser 9 of **synapsin I**.
  For both kinases, hydrophobic residues Leu or Phe at the 5 position, are well tolerated.
  . . . non hydrophobic residues Arg, Ala, or Asp decrease Vmax Km by 55 to >4000 fold.
  At the 3 position, substitution of Ala for Arg leads to decreases of 99 and 343 fold in Vmax Km for **CaMKIV** and CaMKIIalpha, respectively.
  For both kinases, the nature of the residues occupying the , and 4 positions exerts relatively little influence on phosphorylation kinetics.
  **CaMKIV** and CaMKIIalpha respond differently to substitutions at the 2 and 1 positions.
  Substitution of Arg at the 2 position with non basic residues Gln or Ala leads to 6 fold decreases in Vmax Km for **CaMKIV**, but 17 28 fold increases for CaMKIIalpha.
  Additionally, peptides containing Leu, Asp, or Ala at the 1 position are phosphorylated with similar efficiencies by **CaMKIV**.
  . . . the Leu substituted peptide is preferred by CaMKIIalpha by a factor of 5.8 9.7 fold .
  Thus, **CaMKIV** and CaMKIIalpha preferentially phosphorylate substrates with the motifs Hyd X Arg X X Ser\* Thr\*, and Hyd X Arg NB X Ser\* Thr\* Hyd, respectively, where Hyd represents a hydrophobic, X any, and NB a non basic amino acid residue.
  The different specificities of the two kinases may contribute to their targeting to distinct physiological substrates during Ca2ion dependent cellular events.
